# Region-Specific Proteomic Profiles of Extracellular Vesicles (EVs) Derived from Human Macular and Peripheral RPE-Choroid Explants

**DOI:** 10.3390/biomedicines14081715

**Published:** 2026-07-30

**Authors:** Jingwen Zeng, Jialing Zhang, James Schulz, Azhar Dzulhadj B. Arafah, Sora Lee, Michelle Yam, Yi Shen, Fanfan Zhou, Ting Zhang, Mark C. Gillies, Ling Zhu

**Affiliations:** 1 Macula Research Group, Save Sight Institute, Faculty of Medicine and Health, The University of Sydney, Sydney 2006, Australia; 2 School of Chemical and Biomolecular Engineering, Faculty of Engineering, The University of Sydney, Sydney 2006, Australia; 3 School of Pharmacy, Faculty of Medicine and Health, The University of Sydney, Sydney 2006, Australia

**Keywords:** extracellular vesicles (EVs), retinal pigment epithelium (RPE)-choroid, proteomics, secretome, macula, periphery, retina, macular disease

## Abstract

**Background:** Regional heterogeneity of the macula and peripheral retinas contributes to the differences in retinal physiology, metabolic demand and susceptibility to macular diseases such as age-related macular degeneration (AMD), diabetic macular oedema (DME) and Macular telangiectasia type 2 (MacTel). Extracellular vesicles (EVs) released from the retinal pigment epithelium (RPE)-choroid are increasingly recognised as mediators of extracellular communication and correlate with the molecular biological states of their tissue of origin. However, regional variation in EV composition in human RPE-Choroid tissues remains poorly characterised. **Methods:** Human macular and peripheral RPE-choroid explants from non-diseased donor eyes (*n* = 4) were cultured ex vivo using a transwell system. EV and EV-depleted conditioned media were collected by differential ultracentrifugation, respectively. EVs were characterised by transmission electron microscopy (TEM) and nanoparticle tracking analysis (NTA). Proteomic profiling was performed using LC-MS/MS followed by multivariate, pathway, and differential expression analyses. **Results:** TEM and NTA confirmed the presence of vesicle-like particles in a 119–140 nm size range, indicating small EVs in both regions. Proteomic analysis demonstrated a region-associated separation between the macular and peripheral samples in both EV proteome and EV-depleted soluble secretome datasets. GO enrichment analysis revealed that macular EV proteomes were enriched for wound healing, cell–substrate adhesion, and focal adhesion-related terms with a high abundance of integrin and annexin family members, whereas peripheral EV proteomes were enriched for retinoid- and vitamin-binding terms. In the EV-depleted soluble secretome, macular-enriched proteins were associated with actin binding and extracellular matrix-related terms, while peripheral-enriched proteins were correlated with RNA localisation and nuclear compartment terms. Comparative analysis identified EV-specific, secretome-specific, and shared extracellular protein pools. Several EV-specific markers and membrane proteins such as annexins and integrins, showed a relatively high enrichment in macular EV proteomes, whereas CD63 is more abundant in peripheral EV proteomes. In contrast, EV-depleted soluble secretome contains cytokine- and ligand-associated proteins, including MIF, SPP1, and IL6, which may suggest that EVs and soluble secreted proteins represent partially distinct extracellular signalling compartments. **Conclusions:** Human macular and peripheral RPE-choroid explants released secretory proteins in a regional differentiated manner, supported by PLS-DA and GO enrichment analyses of both EV proteomes and EV-depleted soluble secretome. Further analysis of the EV proteomes may show that such differences were also able to be reflected in protein categories such as EV-specific markers, mitochondrial and membrane proteins. These findings provide a foundation for future investigations into the role of EV-mediated communication in macular diseases and may support the development of region-specific extracellular biomarkers and therapeutic targets.

## 1. Introduction

The retinal pigment epithelium (RPE) is a highly specialised monolayer of pigmented cells that plays an essential role in maintaining retinal structure and function. Positioned between the photoreceptors and the choroid, the RPE regulates nutrient transport, phagocytoses the photoreceptor outer segments, and maintains the integrity of the outer blood–retinal barrier [1,2,3]. Disruption of RPE homeostasis is a central feature of several retinal diseases, most notably age-related macular degeneration (AMD), a leading cause of irreversible vision loss worldwide [4]. Macular diseases such as AMD, diabetic macular oedema (DME) and Macular telangiectasia type 2 (MacTel) predominantly affect the macula, resulting in progressive central vision impairment and markedly impacting on quality of life [5,6,7,8].

The human retina has a pronounced regional heterogeneity, particularly between the macula and peripheral retinas. The macula is a small central region responsible for high-acuity vision and colour perception, characterised by a high density of cone photoreceptors and substantial metabolic demand. While the fovea is cone-exclusive, the macula region contains both rod and cone photoreceptors, with a higher cone-to-rod ratio than the peripheral regions [9,10]. Due to its elevated metabolic activities and specialised photoreceptor composition, the macula is more vulnerable to oxidative stress and degeneration [7,11]. These structural and functional differences are accompanied by distinct metabolic profiles and stress susceptibilities.

Extracellular vesicles (EVs) are membrane-bound nanoparticles released by cells into the extracellular environment and are increasingly recognised as the key mediators of intercellular communication [12,13,14]. EVs comprise a heterogeneous population, including exosomes (typically ~30–150 nm in diameter) and microvesicles (approximately 100–1000 nm) [15,16], and carry a diverse cargo of proteins, lipids and nucleic acids reflective of their cell of origin [17,18,19]. EVs in the retina have been implicated in local signalling processes, cellular stress responses, and the maintenance of tissue homeostasis. In particular, RPE-derived EVs may contribute to the communication in the outer retina and may provide insights into cellular states and their molecular composition [4,17].

In addition to their role in intercellular communication, EVs are actively secreted by RPE cells through tightly regulated cellular processes. As a polarised epithelial layer, the RPE releases EVs via the endosomal pathway, where intraluminal vesicles are formed within multivesicular bodies (MVBs) and subsequently secreted as exosomes upon fusion of MVBs with the plasma membrane [18,20,21]. Under pathological conditions associated with macular diseases, EVs secreted by RPE cells may be altered in both quantity and composition. Oxidative stress and chronic inflammation, which are implicated in several macular diseases, have been shown to increase EV release and modify EV cargo, including proteins associated with cellular stress responses, complement activation, and cytoskeletal regulation [22,23]. In addition, stress-induced shedding of adhesion-related proteins via EVs may compromise RPE structural integrity and contribute to retinal dysfunction. EVs derived from stressed RPE cells have been proposed to propagate pathological signals to neighbouring cells, thereby amplifying local damage within the retinal microenvironment [11].

Despite increasing interests in retinal EV research, most studies focus on cultured RPE cell models or its disease models, with a limited attention to physiologically relevant tissue systems or regional variation [24]. In particular, the proteomic composition of EVs derived from macular and peripheral human RPE-choroid tissues has not been investigated before. To fill in the research gap, the current study characterises and compares the proteomic profiles of EVs released from separately cultured human macular and peripheral RPE-choroid explants.

## 2. Materials and Methods

### 2.1. Human Donor-Tissue Collection and RPE-Choroid Explant Culture

Human donor eyes without known ocular disease (<48 h post mortem) were obtained from the New South Wales Lions Eye Bank, Sydney, NSW, Australia. One eye was used per donor (*n* = 4 donors) and paired macular and peripheral RPE-choroid explants were prepared from each eye, giving eight explant samples (four macular and four peripheral) in total. Following corneal excision for transplantation, the remaining posterior eyecups were processed for tissue collection. The neural retina was carefully removed, and RPE-choroid explants were isolated by microdissection. Paired samples from the macular and peripheral regions were obtained using 8 mm biopsy punches (Cat# BP-80F, Kai Medical, Seki, Japan) from freshly dissected tissue. Although the anatomical macula is approximately 5.5 mm in diameter, an 8 mm punch was selected to ensure reliable preservation of the central macular region, as the edge of the RPE-choroid tissue may detach during separation of the choroid from the sclera. Each explant was transferred to a 12-well plate (Cat# 3513, Corning Incorporated, Corning, NY, USA) containing polyester membrane inserts (12 mm, 0.4 µm; Cat# CLS3460, Corning). Explants were cultured in Dulbecco’s Modified Eagle Medium/Nutrient Mixture F-12 (DMEM/F-12; Cat# 11320033, Gibco, Grand Island, NY, USA) supplemented with 10% EV-depleted foetal bovine serum (FBS; Cat# F9423, Sigma-Aldrich, St. Louis, MO, USA) and maintained at 37 °C in a humidified incubator with 5% CO_2_ for 3 days prior to medium collection. The EV-depleted FBS was prepared by ultracentrifugation to remove extracellular vesicles and subsequently filtered through a 0.22 µm filter.

### 2.2. Conditioned-Medium Collection

Conditioned medium was collected from RPE-Choroid explants cultured in transwell inserts for 3 days. Culture medium was replaced every 24 h with fresh DMEM/F-12 supplemented with 10% EV-depleted FBS. Conditioned medium from the upper and lower chambers was collected at each time point and pooled separately for macular and peripheral samples. Samples were transferred into autoclaved 1.7 mL microcentrifuge tubes (Cat# MCT-150-C, Axygen, Union City, CA, USA) and stored at 4 °C until further processing.

### 2.3. Extracellular Vesicle Isolation by Differential Centrifugation

EVs were isolated from conditioned medium by differential centrifugation. Macular and peripheral samples were processed separately throughout. Cell debris and large vesicles were removed by sequential centrifugation at 300× *g* for 10 min, 2000× *g* for 20 min, and 10,000× *g* for 30 min at 4 °C using an Eppendorf 5424 R centrifuge (Eppendorf SE, Hamburg, Germany). The clarified supernatant was then transferred to ultracentrifuge tubes (Cat# 331372, Beckman Coulter, Brea, CA, USA; 13.2 mL thinwall polypropylene tubes, 14 × 89 mm) and subjected to ultracentrifugation at 100,000× *g* for 70 min at 4 °C using an ultracentrifuge (Optima XPN-100, Beckman Coulter) equipped with an SW41 Ti rotor. The EV-depleted soluble supernatant was collected separately, and the EV pellet was washed with ice-cold phosphate-buffered saline (PBS; Cat# 09-2051-100, Astral Scientific, Taren Point, NSW, Australia). PBS used for washing was double filtered through 0.22 µm and 0.025 µm filters. The washed pellet was ultracentrifuged again at 100,000× *g* for 70 min at 4 °C, and the final EV pellet was resuspended in EV-free filtered PBS for storage at −80 °C or immediate characterisation.

### 2.4. Transmission Electron Microscopy (TEM)

The carbon-coated copper grids (300 mesh; Cat# EMSCFT300-CU-50, ProSciTech, Kirwan, QLD, Australia) were glow-discharged using a glow-discharge system (Quorum Technologies, Lewes, UK, GlowCube) before sample loading. EV samples were adsorbed onto the grids, negatively stained with 2% (*w*/*v*) uranyl acetate (UA), and air-dried. Grids were examined using a Tecnai 12 transmission electron microscope (Thermofisher, Waltham, MA, USA) operated at 120 kV at the Sydney Microscopy & Microanalysis facility. Digital images were acquired using a complementary metal-oxide semiconductor (CMOS) camera (Rio9, Gatan, Pleasanton, CA, USA) and processed using Digital Micrograph software (version 3.62; Gatan). Scale bars correspond to 500 nm. Prepared grids were stored in a grid storage box (Cat# HL066, ProSciTech).

### 2.5. Nanoparticle Tracking Analysis (NTA)

EV particle size distribution and concentration were measured by a nanoparticle tracking analyser (PMX-430-12J-R5, Particle Metrix, Inning am Ammersee, Germany) at the Sydney Analytical facility. Samples were diluted 1:2000 in EV-free filtered PBS, prior to analysis. Measurements were performed at 25 °C using ZetaView software (version 8.05.16 SP3) with a 488 nm laser. Video acquisition was conducted with a camera sensitivity of 80 and shutter speed of 100, and particle tracking was performed across 11 positions within the measurement cell. Size distributions are reported as mean ± SD across these capture positions.

### 2.6. Lactate Dehydrogenase (LDH) Release Assay

LDH activity was measured using the CyQUANT™ LDH Cytotoxicity assay kit (Cat# C20300, Thermofisher) following the manufacturer’s protocol. RPE-choroid explants were cultured for 3 days. On each day, the culture medium was refreshed (with a single medium wash), the explants were incubated for 1 h, and 50 µL of conditioned medium was collected before the explants were returned to culture; this was repeated on days 1, 2 and 3. The LDH assay was performed on conditioned medium, following the manufacture’s protocol, and absorbance was measured at 492 and 680 nm on a CLARIOstar Plus microplate reader (Cat# 430-501S-FLA, BMG LABTECH, Ortenberg, Germany).

### 2.7. Sample Preparation for Proteomics (Protein Extraction and Digestion)

EV samples were thawed on ice and lysed with 4% (*w*/*v*) sodium deoxycholate (SDC) lysis buffer prepared in 50 mM ammonium bicarbonate (ABC), followed by sonication, to facilitate vesicle disruption. Proteins were precipitated with ice-cold acetone (≥99.5%, Sigma) at −30 °C overnight. After precipitation, samples were centrifuged at 9000× *g* for 20 min at 4 °C and the resulting EV protein pellets were air-dried at room temperature.

EV-depleted soluble supernatant samples, representing the secretome fraction, were mixed with cold methanol and processed without an additional centrifugation step before solubilisation. EV pellets were resuspended in 50 µL of 6 M urea/2 M thiourea solution (Sigma), whereas EV-depleted soluble supernatant samples were resuspended in 100 µL of the same solution. Proteins were reduced with 100 mM DL-dithiothreitol (DTT; Sigma) and alkylated with 250 mM iodoacetamide (IAA; Sigma). For EV pellets, 5 µL of DTT and 5 µL of IAA were used, while EV-depleted soluble supernatant samples were treated with 10 µL of DTT and 10 µL of IAA. Protein concentration was determined using the Qubit™ Protein Assay Kit (Thermofisher), according to the manufacturer’s instructions. Samples were diluted with 50 mM ABC and aliquots containing 1 µg of protein were digested with sequencing-grade trypsin (Promega, Madison, WI, USA) at 1:20 trypsin-to-mass ratio overnight (~16 h) at 37 °C with agitation (digestion mode: 800 rpm). The resulting peptides were acidified to final concentration of 0.1–1% using a 10% (*v*/*v*) trifluoroacetic acid (TFA), and purified using C18 ZipTips (Millipore, Burlington, MA, USA) with acetonitrile (ACN)-containing TFA buffers prepared using ACN (Cat# 34851-4, Sigma-Aldrich). Purified peptides were dried by freeze-drying using an Alpha 2–4 LSCbasic freeze dryer (CHRIST, Osterode am Harz, Germany) and stored at −30 °C until liquid chromatography–tandem mass spectrometry (LC-MS/MS) analysis.

### 2.8. Proteomic Analysis by Liquid Chromatography–Tandem Mass Spectrometry (LC-MS/MS)

Peptide samples were resuspended in proteomics loading buffer containing 3% ACN, sonicated, and centrifuged at 16,000× *g* for 10 min at 4 °C to remove insoluble material. Clarified samples were transferred to a 96-well plate (Cat# AB-0800-L, Thermofisher) and analysed by nano-flow liquid chromatography–tandem mass spectrometry (nanoLC-MS/MS) at the Sydney Mass Spectrometry facility, using an ultra-high-performance liquid chromatography (UHPLC) system (Ultimate 3000, Thermofisher) coupled to a Q Exactive HFX Orbitrap mass spectrometer (Thermofisher).

Peptides were separated on an in-house packed reversed-phase (RP) C18 column packed with 1.9 µm C18 particles (Dr. Maisch GmbH, Ammerbuch, Germany, Pur 120 C18, Cat# r119.aq), maintained at 60 °C and operated at flow rate of approximately 250–300 nL/min, resulting in a backpressure of ~500–600 bar. Mobile phases consisted of buffer A (0.1% formic acid in Optima LC/MS-grade water; Cat# 85178, W6-4, Thermofisher) and buffer B (80% ACN, 19.9% water, 0.1% formic acid). Samples were loaded using buffer containing 3% ACN and 0.1% formic acid with 96.9% of water. Peptides were separated using a 90 min LC gradient, prior to mass spectrometric analysis.

The mass spectrometer was operated in positive-ion mode using nano-electrospray ionisation. Data were acquired in data-independent acquisition (DIA) mode with full MS scans at 70,000 resolution (at *m*/*z* 200) over mass range of *m*/*z* 350–1550, followed by MS/MS scans at 17,500 resolutions. Key acquisition parameters included an automatic gain control (AGC) target of 3 × 10^6^ for MS1 and 1 × 10^5^ for MS2, a 1.2 *m*/*z* isolation window, and a normalised collision energy of 27. System performance and reproducibility were monitored using 30 fmol BSA digest (Cat# P8108S, GeneSearch, Arundel, QLD, Australia) injected at the beginning and end of each sequence, and raw data were acquired using Xcalibur (version 4.1.31.9).

### 2.9. Proteomics Data Analysis

Raw mass spectrometry data were processed using Spectronaut v20 software (Version 20.5.260227.92449, Biognosys, Schlieren, Switzerland) employing a library-free data-independent acquisition (directDIA) workflow. Protein identification was performed by searching against the UniProt Homo sapiens database. False discovery rate (FDR) was controlled at 1% at both peptide and protein levels. Quantification was based on MS2 signal intensities using the default peak area-based approach implemented in Spectronaut. Data were normalized using the local normalization algorithm to correct for systematic technical variation across samples.

### 2.10. Downshifted Gaussian Imputation

Protein abundance data were first filtered to remove proteins with missing or low-confidence values, followed by missing value imputation. Data processing steps were assisted using computational tools. Missing values (NaN) in the proteomics dataset were imputed using a downshifted Gaussian distribution approach, where missing values were replaced with random numbers drawn from a normal distribution (downshift = 1.8, width = 0.3) to simulate low-abundance protein signals [25,26,27,28]. The original and imputed datasets, together with missingness percentages for the EV proteome and EV-depleted soluble secretome, are provided in Supplementary Table S6, Table S7 and Table S8, respectively.

### 2.11. Gene Ontology (GO) Pathway Analysis and Kyoto Encyclopedia of Genes and Genomes Gene-Set Enrichment Analysis (KEGG-GSEA)

GO enrichment analysis was performed separately for proteins enriched in macular and peripheral samples using the clusterProfiler and org.Hs.eg.db packages in R (Rstudio, version 2025.09.2+418). Proteins were considered differentially abundant at a log_2_ fold change threshold of ±0.263 (equivalent to 1.2-fold change), *p*-value < 0.1, *q*-value < 0.2, and gene ratio > 0.1. Enrichment analysis was conducted across all three GO ontologies, including Biological Process (BP), Molecular Function (MF), and Cellular Component (CC) for Homo sapiens. The top 10 pathways per ontology were ranked by *p*-value.

To complement the threshold-based GO analysis with a cut-off-free approach, Kyoto Encyclopedia of Genes and Genomes (KEGG) gene-set enrichment analysis (GSEA) was performed on the full ranked list of all quantified proteins, ranked by log_2_ fold change (clusterProfiler); normalized enrichment scores (NESs) are shown on the *x*-axis.

### 2.12. Partial Least Squares Discriminant Analysis (PLS-DA)

Protein intensities were log_2_-transformed and scaled prior to modelling, with the eight samples as observations and proteins as variables. PLS-DA was fitted with two predictive components, comparing the macular and peripheral groups. The proportion of variance explained by each component is indicated on the score-plot axes, and grouping ellipses are shown as a visual aid to summarise the sample distribution within each region. To assess whether the observed PLS-DA separation exceeded chance, permutation testing was performed using the mixOmics package with 1000 random label permutations, evaluating the between-/within-group separation statistic under leave-one-out cross-validation. Both analyses were performed in R using the ropls and mixOmics packages.

### 2.13. Statistical Analysis

Extracellular vesicle NTA size distribution was presented as mean ± standard deviation (SD), and visualised using GraphPad Prism 11. Proteomic data analysis and visualisation, including PLS-DA, GO pathway, Volcano plots, Venn diagrams, diverging bar plots and KEGG-GSEA were performed in R. All *p*-values are from two-tailed paired *t*-tests.

Protein classification was performed using publicly available databases. Mitochondrial proteins were annotated based on the human MitoCarta3.0 inventory [29], which contains 1136 mitochondrial proteins. Membrane proteins were annotated based on the Human Protein Atlas membrane proteome dataset [30,31], which contains 5339 predicted human membrane proteins. The top 100 EV-specific markers were identified using Vesiclepedia [15], with vesicle marker categories informed by MISEV2023 [19]. 

## 3. Results

### 3.1. Isolation and Characterisation of EVs from Human RPE-Choroid Explants

**Figure 1:**
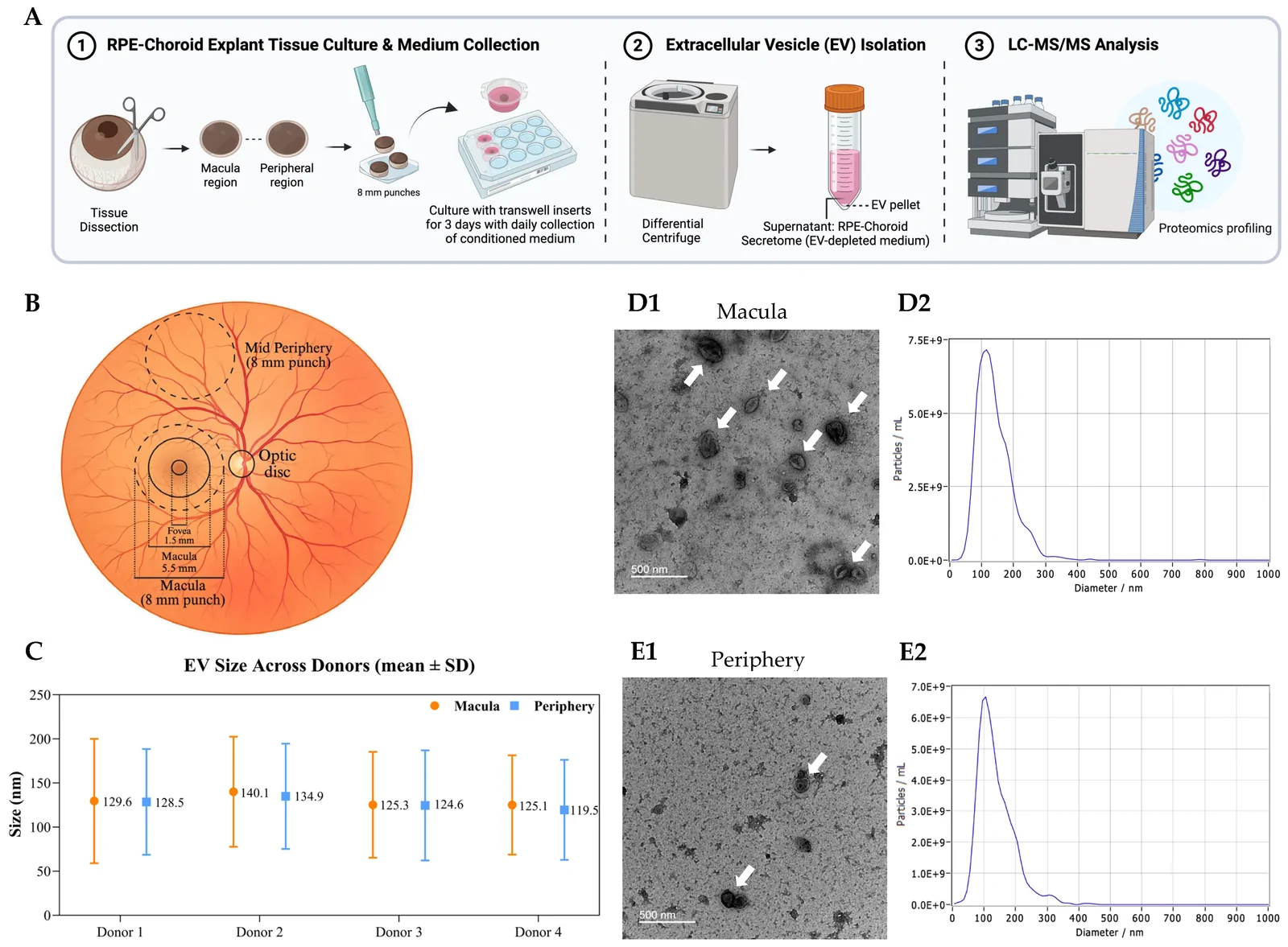
**Isolation and characterization of EVs derived from human RPE-choroid explants.** (**A**) Schematic overview of the experimental workflow. (**B**) Schematic illustration of retinal regions, indicating the macula, fovea, optic disc, and mid peripheral region; 8 mm punches were obtained from macular and peripheral areas. (**C**) EV size distribution in macula (orange) and periphery (blue) across individual donors measured by NTA, presented as mean ± standard deviation (SD). (*n* = 4 donors). (**D1**,**E1**) TEM images of EVs isolated from macular (**D1**) and peripheral (**E1**). RPE-choroid explants exhibited typical vesicle morphology, with white arrows indicating EVs. Scale bar: 500 nm. Corresponding NTA profiles (**D2**,**E2**) showed that most vesicles were within the expected size distribution of EVs. Figure 1A,B were created in BioRender. Zeng, J. (2026). https://BioRender.com/3atnjp2 (accessed on 24 July 2026).

To establish a human tissue-based system for profiling region-specific EVs, macular and peripheral RPE-choroid explants were isolated from non-diseased donor eyes (Figure 1A), with donor information provided in Supplementary Table S1, enabling paired comparison of EVs from anatomically distinct regions of the same donor. Tissue was collected from macular and mid-peripheral regions, as shown in Figure 1B. An LDH assay was performed as an additional quality-control measure to assess cytotoxicity during explant culture, as shown in Supplementary Figure S1C.

TEM and NTA characterised EV preparations isolated by differential centrifugation from both regions. TEM is commonly used for direct visual assessment of EV morphology at nanometre resolution, while NTA provides particle concentration and size distribution. To assess donor-to-donor consistency, NTA-derived EV size measurement was performed across all the paired macular and peripheral samples obtained from four healthy donors (Figure 1C). Mean particle diameters ranged from approximately 119.5 to 140.1 nm across the eight samples, with broadly comparable size distributions in both regions. The size distribution, reflected by the SD, was predominantly below 200 nm across all samples.

Representative TEM images from one donor confirmed the presence of membrane-bound vesicle particles and bilayer structure in both macular and peripheral samples, displaying characteristic round-to-oval morphology (Figure 1(D1,E1)). EV-like particles were observed in samples isolated from both regions. Corresponding NTA profiles demonstrated a predominant particle concentration with peak diameters of approximately ~50 to 200 nm in both regions (Figure 1(D2,E2)). In both regions, the size of most particles was below 200 nm. A minor secondary population was observed at approximately 200–300 nm in both regions, possibly corresponding to larger EVs such as microvesicles. Particle counts were minimal beyond 400 nm.

### 3.2. Comparative Proteomic Analysis of Macular and Peripheral RPE-Derived EV Proteome and EV-Depleted Soluble Secretome

Proteomic profiling was performed on both EV proteome and EV-depleted soluble secretome derived from macular (M) and peripheral (P) RPE-choroid explants. Comparative analyses were conducted using multivariate, pathway, and differential expression approaches.

**Figure 2:**
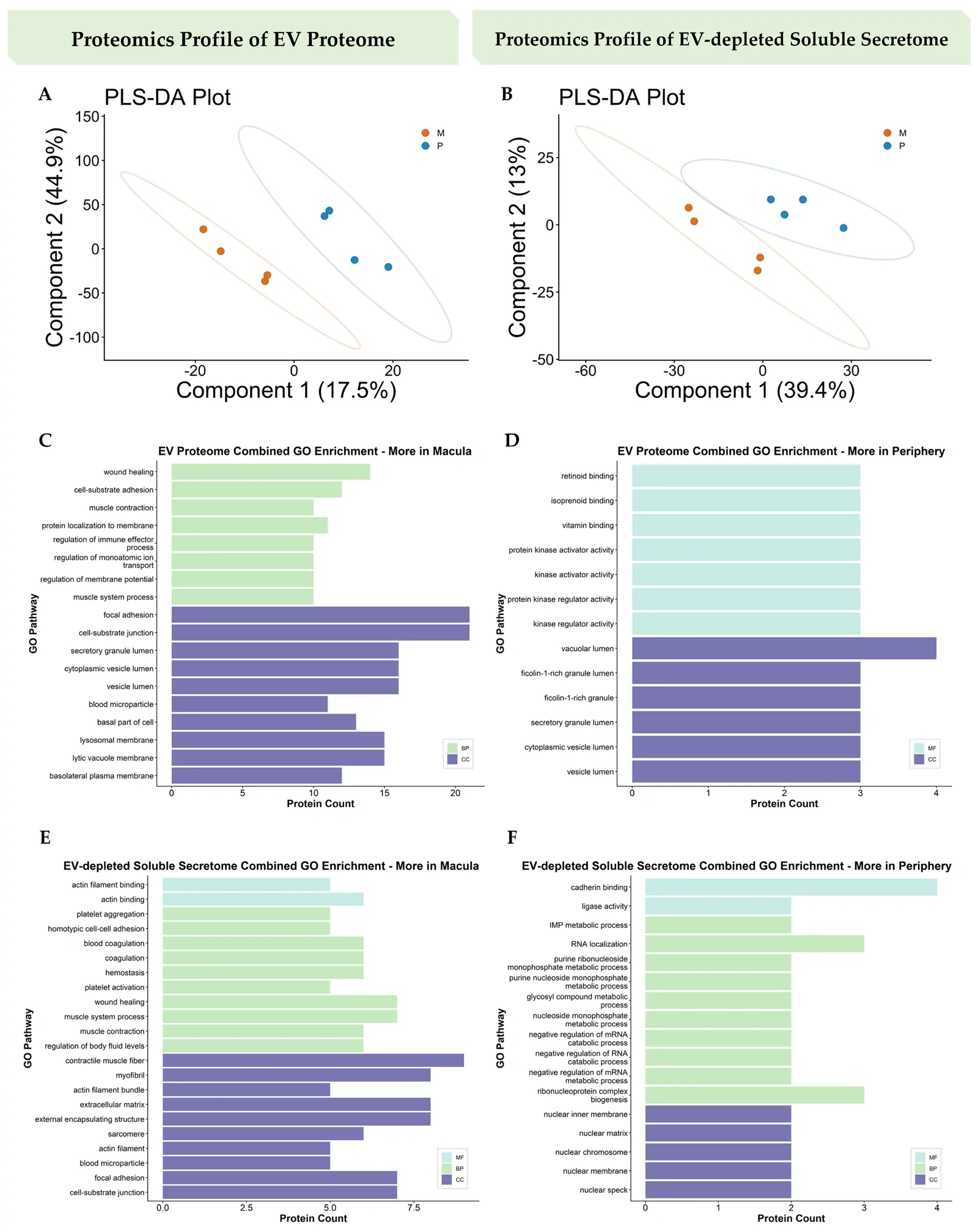

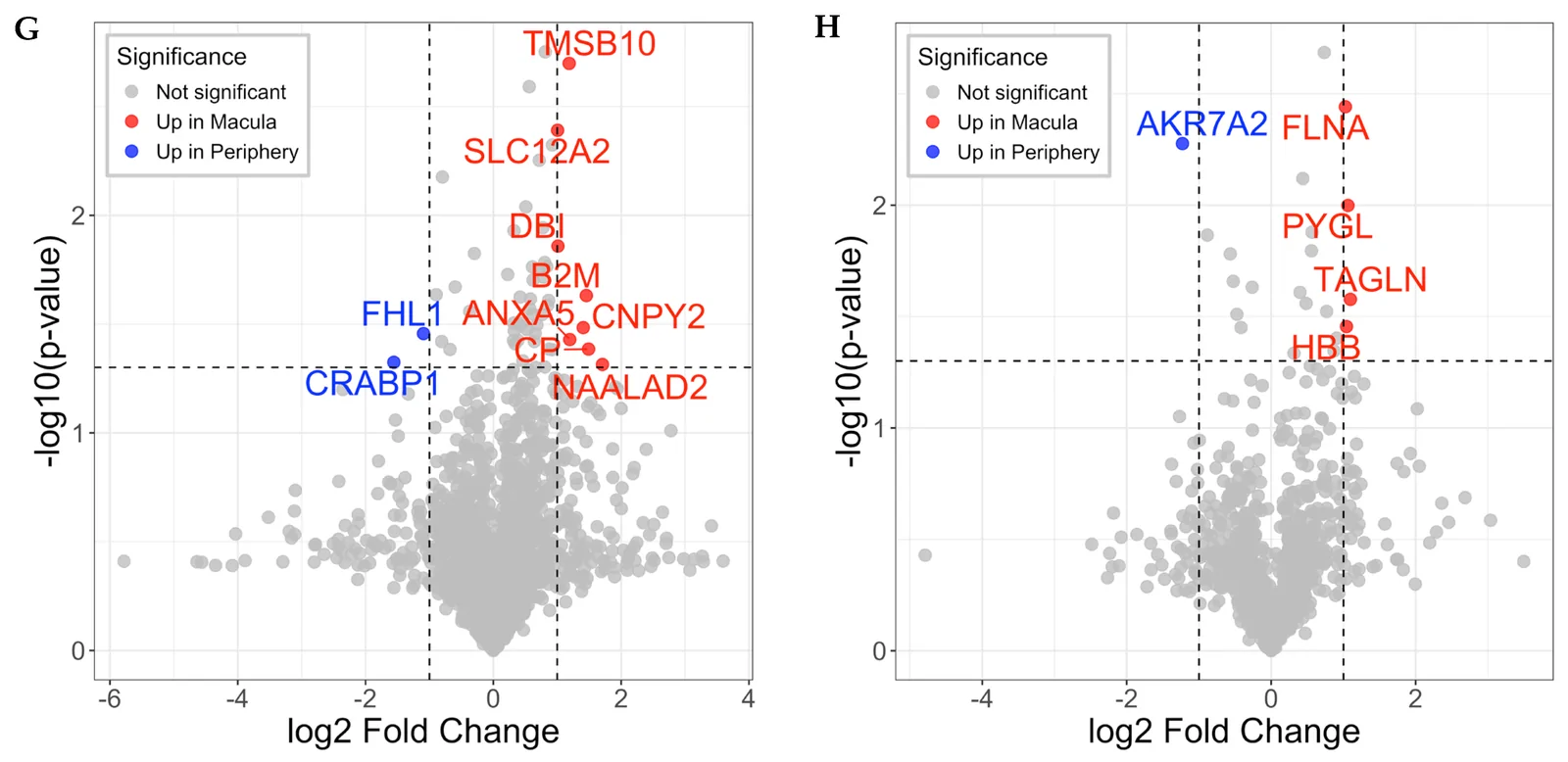
**Comparative proteomic profiling of EV proteomes and EV-depleted soluble secretome derived from human macular and peripheral RPE-choroid explants.** (**A**,**B**) Partial least squares discriminant analysis (PLS-DA) plots of proteomic data from EV proteomes (**A**) and EV-depleted soluble secretome (**B**), showing macular (M) and peripheral (P) groups along Component 1 (%) and Component 2. Grouping ellipses indicate the distribution of samples within each group. (**C**–**F**) GO terms are categorised by ontology: Biological Process (BP, green), Molecular Function (MF, blue), and Cellular Component (CC, purple), with protein count shown on the *x*-axis. (**C**,**D**) GO enrichment analysis of EV proteome proteomic data showing the top enriched GO terms for proteins with higher abundance in macular (**C**) and peripheral (**D**) samples. (**E**,**F**) GO enrichment analysis of EV-depleted soluble secretome proteomic data showing the top enriched GO terms for proteins with higher abundance in macular (**E**) and peripheral (**F**) samples. (**G**,**H**) Volcano plots of differential protein expression of the macular and peripheral samples for EV proteome (**G**) and EV-depleted soluble secretome (**H**). Proteins upregulated in macular samples are shown in red (Log_2_ fold change > 1), those upregulated in peripheral samples are shown in blue (Log_2_ fold change < −1), and non-significant proteins are shown in grey. *p*-value < 0.05. Vertical dashed lines indicate the log_2_ fold change threshold (±1), and the horizontal dashed line indicates the *p*-value threshold (*p* = 0.05).

In the EV proteome proteomics dataset, PLS-DA revealed a separation between the macular (M) and peripheral (P) samples (Figure 2A). Component 1 accounted for 17.5% of the variance and Component 2 accounted for 44.9% of the variance. Grouping ellipses, drawn as a visual aid to summarise the sample distribution within each region, were non-overlapping between macular and peripheral samples. Similarly, a comparable separation of the EV-depleted soluble secretome was observed between macular and peripheral samples (Figure 2B), although the structure of variance was different. Component 1 accounted for 39.4% of the variance and Component 2 for 13.0%, with more variance explained by Component 1; the grouping ellipses of the two groups showed a slight overlap. In addition, permutation testing was performed to assess whether the observed separation between macular and peripheral samples exceeded chance, as shown in Supplementary Figure S1(A1,A2). Given the limited sample size and permutation results, the PLS-DA findings were interpreted as exploratory visualisations, rather than validated classification models.

In the EV proteome proteomics dataset, GO enrichment analysis identified biological processes (BPs), molecular functions (MFs), and cellular components (CCs) associated with regional protein differences (Figure 2C,D). Among proteins with higher abundance in macular EVs, enriched BP terms included wound healing, cell–substrate adhesion, regulation of immune effector processes, and regulation of membrane potentials, while CC terms included focal adhesion, cell–substrate junction, vesicle lumen, lysosomal membrane, and basolateral plasma membrane (Figure 2C). Among proteins with higher abundance in peripheral EVs, enriched MF terms included retinoid binding, vitamin binding, and kinase regulator activity, while CC terms included vacuolar lumen, ficollin-1-rich granule lumen, and secretory granule lumen (Figure 2D). In the EV-depleted soluble secretome dataset, GO enrichment analysis revealed a distinct set of enriched terms (Figure 2E,F). Among proteins with higher abundance in the macular secretome, enriched BP terms included blood coagulation, haemostasis, platelet aggregation, wound healing, and muscle contraction, MF terms included actin binding and actin filament binding, and CC terms included extracellular matrix, actin filament bundle, contractile muscle fibre, focal adhesion, and blood microparticle (Figure 2E). Among proteins with higher abundance in the peripheral secretome, enriched BP terms included purine nucleotide metabolic processes, RNA localisation, and negative regulation of mRNA catabolism, MF terms included cadherin binding and ligase activity, and CC terms included nuclear membrane, nuclear matrix, nuclear speck, and ribonucleoprotein complex (Figure 2F). The corresponding GO pathway information is provided in Supplementary Tables S2 and S3. In addition, cut-off-free KEGG-GSEA using the full ranked protein list was performed to support pathway-level trends. Several KEGG pathways were consistent with the pathway trends observed in GO enrichment analysis, with results shown in Supplementary Figure S1(B1,B2), and corresponding pathway information provided in Supplementary Tables S4 and S5.

To identify specific proteins differentially expressed between the macular and peripheral regions, volcano plots were generated for both EV proteome and EV-depleted soluble secretome. In the EV proteome dataset, a number of proteins showed nominal differential abundance between the paired macular and peripheral samples (Figure 2G). The most nominally upregulated proteins in the macular EVs included TMSB10, SLC12A2, DBI, B2M, ANXA5, CNPY2, CP, and NAALAD2. In contrast, proteins upregulated in the peripheral EVs included FHL1 and CRABP1, with CRABP1 showing the greatest fold change toward the peripheral direction. In the EV-depleted soluble secretome proteomics dataset, proteins with higher abundance upregulated in the macular region included FLNA, PYGL, TAGLN, and HBB, while AKR7A2 with higher abundance was upregulated in the peripheral region (Figure 2H).

### 3.3. Distinct Protein Signatures Identified in EV Proteome and EV-Depleted Soluble Secretome

**Figure 3:**
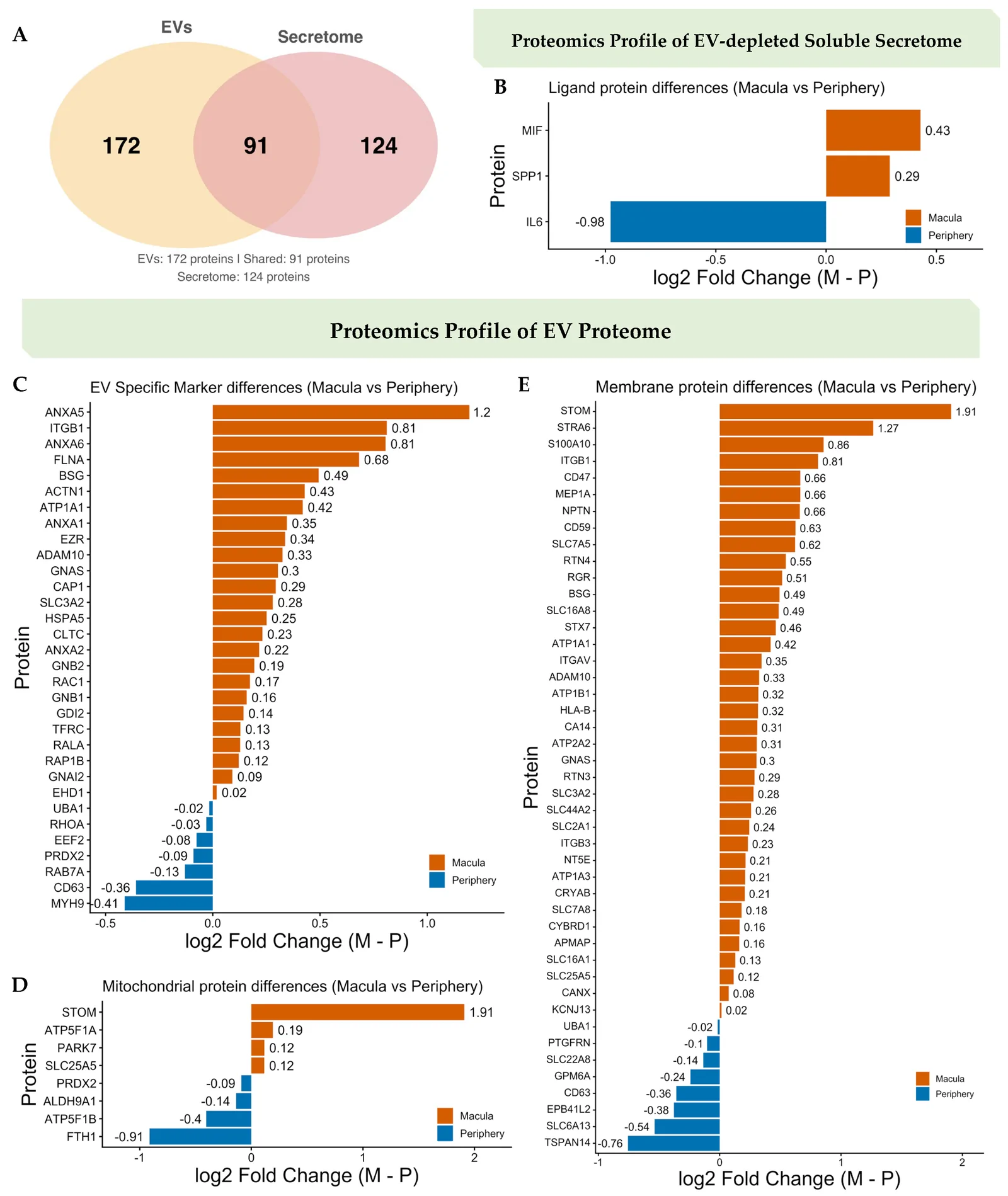
**Differential proteomic analysis of EV proteome and EV-depleted soluble secretome in the paired macular and peripheral samples.** (**A**) Venn diagram showing the distribution of proteins identified in EV proteome and EV-depleted soluble secretome. Only proteins with valid quantitative values (no missing values) and abundance greater than 1000 were included. (**B**) Diverging horizontal bar chart showing the differential expression of ligand proteins in the secretome of the paired macular and peripheral samples. (**C**–**E**) Diverging horizontal bar charts showing the differential expression of EV protein categories in the paired macular and peripheral samples, including EV specific markers (**C**), mitochondrial proteins (**D**), and membrane proteins (**E**). For (**B**–**E**), bars represent log_2_ fold change (Macula–Periphery), with positive values indicating higher abundance in macular samples and negative values indicating higher abundance in peripheral samples.

To characterise the proteomic landscape of EV proteome and EV-depleted soluble secretome, proteins identified in both fractions were compared. The Venn diagram revealed a total of 387 proteins identified in both fractions, comprising 172 EV-specific proteins, 124 secretome-specific proteins, and 91 shared proteins (Figure 3A). Subsequent analyses focused on those proteins uniquely identified in both compartments.

To further characterise the 172 EV-specific proteins, subsets of biologically relevant protein categories were examined, including EV-specific markers, mitochondrial proteins and membrane proteins.

To validate the identity of the isolated particles and assess region-specific differences in EV marker expression, well-established EV marker proteins were identified within the 172 EV-specific proteins (Figure 3C). Most EV markers were enriched in the macular region, including annexin family members (ANXA1, ANXA2, ANXA5, and ANXA6), integrins (ITGB1), heat shock proteins (HSPA5), and cytoskeletal proteins (FLNA and ACTN1), with ANXA5 showing the highest macular enrichment. In contrast, a smaller subset of EV markers was upregulated in the peripheral region, most notably CD63 and MYH9.

A subset of the 172 EV-specific proteins was identified as mitochondrial proteins, with differential expression observed between macular and peripheral EV fractions (Figure 3D). STOM showed the highest enrichment in the macular region, followed by ATP5F1A, PARK7, and SLC25A5. Mitochondrial proteins upregulated in the peripheral EV fraction mainly included FTH1, ATP5F1B, ALDH9A1, and PRDX2.

Among the 172 EV-specific proteins, a substantial proportion were identified as membrane-associated proteins, consistent with the membrane-bound vesicles (Figure 3E). The identified membrane proteins spanned across several functionally distinct categories, including CD family transmembrane proteins (e.g., CD47, CD59, CD63), solute carrier transporters (SLCs), integrins (e.g., ITGB1, ITGAV), ATPases (e.g., ATP1A1, ATP1B1, ATP2A2), and TMEM-associated proteins. There were 37 membrane proteins showed higher abundance in the macular EVs, with STOM and STRA6 showing the greatest macular enrichment, while a smaller subset including TSPAN14 and CD63 was predominantly upregulated in the peripheral region.

## 4. Discussion

This study provides an exploratory comparison of EV proteome and EV-depleted soluble secretome profiles released from paired human macular and peripheral RPE-choroid explants. In the EV proteome dataset, PLS-DA showed a macular–peripheral distribution pattern, although this should be interpreted cautiously, given the small sample size, within-group variability, and the exploratory nature of the model (Figure 2A). The separation was observed mainly along Component 2, while Component 1 may reflect non-region-related variation, including inter-donor variability. The EV-depleted soluble secretome showed a less-distinct distribution pattern, with partial overlap between grouping ellipses (Figure 2B). Together with the protein-category and pathway-level analyses, these observations could suggest that EV proteome and EV-depleted soluble secretome profiles capture partially distinct aspects of regional extracellular molecular variation in human RPE-choroid explants [9,32,33].

GO enrichment analysis could provide further insights into the regional biological differences between macular and peripheral EV proteome and EV-depleted soluble secretome (Figure 2C–F; Supplementary Tables S2 and S3). In the EV proteome, macular-enriched proteins were highlighted by integrin and annexin family members including ITGB1, CD44, ANXA5, and CD59 (Figure 2C). Annexins and integrins are established components of RPE biology. ANXA5 has been shown to regulate αvβ5 integrin at the apical phagocytic surface of RPE cells, while integrin-mediated focal adhesion kinase signalling is essential for photoreceptor outer-segment clearance [34,35]. Their enrichment in macular EVs may reflect region-associated differences in phagocytosis-related and adhesion-associated EV cargo. In contrast, peripheral-enriched proteins were associated with retinoid- and vitamin-binding MF terms, represented by RHO, CRABP1, and FABP5 (Figure 2D). It is known that the RPE plays a central role in retinoid metabolism through the visual cycle, processing all-trans-retinol to regenerate the visual chromophore 11-cis-retinal for photoreceptors [36,37]. The enrichment of retinoid- and vitamin-binding terms in peripheral could indicate region-associated differences in retinoid-related cargo. In the EV-depleted soluble secretome, macular-enriched proteins were linked to the RPE cytoskeleton, including actin filaments and associated proteins like filamin A (Figure 2E), maintaining a functional association with the extracellular matrix indispensable to RPE integrity and photoreceptor support [38]. In contrast, peripheral-enriched proteins were correlated with RNA localisation and nucleoside monophosphate metabolic processes, alongside nuclear compartment terms (Figure 2F). Notably, these pathway patterns support the possibility that macular and peripheral RPE-choroid explants release extracellular proteins with regionally distinct biological themes, rather than simply differing in total protein abundance.

Volcano plot analysis provided additional protein-level support for the regional patterns observed in the pathway analyses (Figure 2G,H). In the EV proteome, TMSB10 belongs to the β-thymosin family and is associated with actin binding, cytoskeletal organisation, and actin dynamics [38]. SLC12A2 encodes the Na-K-Cl cotransporter NKCC1, a membrane transporter involved in coupled sodium, potassium, and chloride movement, as well as cellular ion homeostasis [39,40,41,42]. In the EV-depleted soluble secretome, FLNA is an actin-binding scaffold protein contributing to cytoskeletal organisation and mechanotransduction [43,44]. PYGL encodes liver glycogen phosphorylase, a key enzyme in glycogen breakdown and glucose metabolism [45]. AKR7A2 belongs to the aldo-keto reductase family, which has established roles in aldehyde and ketone reduction, detoxification, and cellular stress-related metabolism [46,47]. The absence of common differentially represented proteins was observed between the EV proteome and EV-depleted soluble secretome datasets, suggesting that EV proteome and EV-depleted soluble secretome proteins represent distinct extracellular protein pools, consistent with the concept that EV cargo is shaped by selective cargo-loading mechanisms, while extracellular protein preparations may also contain non-vesicular extracellular components [24,48].

The comparison between EV proteome and EV-depleted soluble secretome identified shared and region-specific protein pools (Figure 3A). Proteins detected in both regions may be derived from multiple extracellular release mechanisms including EV-associated release and non-vesicular extracellular protein complexes, although their precise origin cannot be resolved from the present dataset [24,49]. Within the secretome-specific fraction, cytokine-associated ligands including MIF, SPP1, and IL6, showed regional differences in abundance (Figure 3B). Their enrichment within the secretome-specific pool suggests that these proteins were more closely associated with soluble extracellular signalling than with EV-enriched cargo, in this dataset. MIF has been associated with immune regulation and oxidative stress responses in human RPE models [50], while SPP1/osteopontin is an extracellular matrix-associated glycoprotein implicated in tissue remodelling and retinal disease-associated extracellular deposits [51]. IL6 is a pleiotropic cytokine involved in retinal inflammation, vascular permeability, and macular oedema-related pathology [52,53]. Overall, these findings may suggest that the secretome captures region-associated soluble cytokine-related signals.

EV-associated proteins such as annexins, integrins, heat shock proteins, cytoskeletal proteins, and the tetraspanin CD63 (Figure 3C), could support the enrichment of EV-associated materials, and complement the morphological and biophysical characterisation shown in Figure 1 [18,19]. Several EV-associated proteins showed regional variation between macular and peripheral EVs. Annexins are calcium-dependent phospholipid-binding proteins involved in membrane organisation and dynamics [54], and integrins mediate cell-extracellular matrix interactions and intracellular signalling. CD63 is a tetraspanin enriched in late endosomal compartments and intraluminal vesicles, which is commonly associated with small-EV or exosome-related pathways [19]. The higher abundance of CD63 in peripheral EVs may, therefore, indicate regional differences in EV membrane composition, endosomal-associated cargo, or relative contribution of CD63-positive EV populations [55,56]. Although the functional implications of these findings remain uncertain, the observed regional variation suggests that differences between macular and peripheral EVs may extend beyond total protein abundance and contribute to subtle differences in EV composition and cargo packaging.

Mitochondrial proteins were also identified in the EV-specific fraction (Figure 3D). The macular RPE-choroid complex exists under uniquely elevated oxidative and bioenergetic pressure, due to cone support, lipid processing, and chronic mitochondrial stress. Enrichment of ATP5F1A in macular EVs may reflect selective export of ATP synthase remodelling intermediates or mitochondrial stress-associated cargo, potentially linked to cristae restructuring, mitovesicle formation, and early degenerative adaptation. In contrast, ATP5F1B enrichment in peripheral EVs may represent lower-stress baseline mitochondrial turnover associated with rod-dominant retinal support. PARK7 was also detected in macular EV cargo, which has been reported to participate in oxidative stress responses and mitochondrial homeostasis in retinal tissues [57]. Together, these findings could suggest that EV cargo may reflect regional differences in membrane-associated transport and metabolic state [58], but not mitochondrial dysfunction in the donor tissues.

Among the EV-specific proteins identified in this study, membrane proteins are the prominent category, including integrins, ATPases, SLCs, TMEM-associated proteins and CD family proteins (Figure 3E) [59]. Several SLC family members were detected among EV proteome membrane proteins, which may support the potential involvement of RPE-choroid EVs in transport-related processes such as nutrient exchange, metabolite handling and ion homeostasis [39,42,60]. Notably, SLC2A1/GLUT1 was enriched in macular EVs, and, given the established role of GLUT1 in glucose transport across the RPE and in supporting photoreceptor energy metabolism, its enrichment may reflect the higher metabolic demands of the macular region [33,61]. Integrins including ITGB1 and ITGAV are relatively enriched in macular EVs and involved in cell-extracellular matrix interactions and intracellular signalling [18,62]. Their regional enrichment may reflect differences in extracellular matrix-associated interactions between the macular and peripheral RPE-choroid tissues. The presence of SLCs and ATPases in EV proteome may indicate the involvement of transport-associated functions at the RPE-choroid interface, such as nutrient exchange and ion homeostasis [3,62,63].

It is noteworthy that there are several limitations to the current study. First, this study included four biological replicates, reflecting the limited but valuable availability of human-donor eye tissue, which may limit statistical power and generalisability. Although paired macular and peripheral comparisons help reduce inter-donor variability, larger donor cohorts may further validate in this study. Secondly, the explant model cannot differentiate apical from basal release. Because conditioned medium from the upper and lower chambers was pooled, the EV proteome and EV-depleted soluble secretome samples contain material released from both retinal-facing and choroid-facing surfaces, as well as from choroidal components within the RPE-choroid complex. Future studies may employ spatially resolved approaches to clarify the directional and cellular origins of these extracellular proteins [12]. Most importantly, this study only included non-diseased donor tissues. Thus, it remains unclear whether the region-specific EV proteome and EV-depleted soluble secretome signatures are altered in macular diseases. It is expected that future studies can include clinically characterised disease donor eyes such as AMD, DME, and MacTel, to determine the association of extracellular proteomic profiles with disease-specific molecular changes or progression [6,8,23]. In addition, the regional mitochondrial protein differences reported here are based on proteomic data; functional mitochondrial assays were not performed, and future studies incorporating functional readouts would be required, to directly assess regional mitochondrial activity.

## 5. Conclusions

The present study demonstrates that human RPE-choroid explants release EV proteome and EV-depleted soluble secretome secreted proteins with region-specific proteomic differences between the macula and periphery retinas. EV cargo showed distinct regional features, including membrane-associated, mitochondrial and EV-related proteins, whereas the EV-depleted soluble secretome captured soluble cytokine-associated signals. To our knowledge, this study provides one of the first region-resolved analyses of EV proteomes and EV-depleted soluble secretome derived from human RPE-choroid tissues, highlighting an extracellular compartment that remains largely unexplored. Future studies using larger cohorts and clinically characterised macular-disease donor eyes are expected to investigate the association of extracellular proteomic signatures and retinal diseases.

## Data Availability

The mass spectrometry proteomics data have been deposited at the ProteomeXchange Consortium (https://proteomecentral.proteomexchange.org; accessed on 1 July 2026) via the iProX partner repository [64,65] with the dataset identifier PXD080419.

## Supplementary Materials

The following supporting information can be downloaded at: https://www.mdpi.com/article/10.3390/biomedicines14081715/s1; Figure S1: (A) Permutation testing of PLS-DA models, (B) KEGG pathway analysis, and (C) LDH cell viability test of the EV proteome and EV-depleted soluble secretome; Table S1: Donor Information; Table S2: EV proteome GO pathway protein list; Table S3: EV-depleted soluble secretome GO pathway protein list; Table S4: KEGG-GSEA pathway EV proteome information list; Table S5: KEGG-GSEA pathway EV-depleted soluble secretome information list; Table S6: EV proteome original and imputation data; Table S7: EV-depleted soluble secretome original and imputation data; Table S8: Dataset missing-value calculation.

## Abbreviations

The following abbreviations are used in this manuscript:

ABC	Ammonium bicarbonate
ACN	Acetonitrile
AGC	Automatic gain control
AMD	Age-related macular degeneration
ANXA	Annexin
BP	Biological Process
BSA	Bovine serum albumin
Cat#	Catalogue number
CC	Cellular Component
CMOS	Complementary metal-oxide semiconductor
CO_2_	Carbon dioxide
DIA	Data-independent acquisition
directDIA	Direct data-independent acquisition
DMEM/F-12	Dulbecco’s Modified Eagle Medium/Nutrient Mixture F-12
DME	Diabetic macular oedema
DTT	DL-dithiothreitol
EV	Extracellular vesicle
FBS	Foetal bovine serum
FDR	False discovery rate
GO	Gene Ontology
IAA	Iodoacetamide
LC-MS/MS	Liquid chromatography–tandem mass spectrometry
MacTel	Macular telangiectasia type 2
M	Macular
MF	Molecular Function
MS	Mass spectrometry
MS/MS	Tandem mass spectrometry
MVB	Multivesicular body
*m*/*z*	Mass-to-charge ratio
NaN	Not a number
nanoLC-MS/MS	Nano-flow liquid chromatography–tandem mass spectrometry
NKCC1	Sodium-potassium-chloride cotransporter 1
NTA	Nanoparticle tracking analysis
P	Peripheral
PBS	Phosphate-buffered saline
PLS-DA	Partial least squares discriminant analysis
RPE	Retinal pigment epithelium
RP	Reversed phase
SD	Standard deviation
SDC	Sodium deoxycholate
SLC	Solute carrier
TEM	Transmission electron microscopy
TFA	Trifluoroacetic acid
TMEM	Transmembrane protein
UA	Uranyl acetate
UHPLC	Ultra-high-performance liquid chromatography
ACTG1	Actin gamma 1
ACTN1	Alpha-actinin 1
AKR7A2	Aldo-keto reductase family 7 member A2
ALDH9A1	Aldehyde dehydrogenase 9 family member A1
ANXA1	Annexin A1
ANXA2	Annexin A2
ANXA5	Annexin A5
ANXA6	Annexin A6
ATP1A1	ATPase Na^+^/K^+^ transporting subunit alpha 1
ATP1B1	ATPase Na^+^/K^+^ transporting subunit beta 1
ATP2A2	ATPase sarcoplasmic/endoplasmic reticulum Ca^2+^ transporting 2
ATP5F1A	ATP synthase F1 subunit alpha
ATP5F1B	ATP synthase F1 subunit beta
B2M	Beta-2-microglobulin
CD44	CD44 molecule
CD47	CD47 molecule
CD59	CD59 molecule
CD63	CD63 molecule
CNPY2	Canopy FGF signalling regulator 2
CP	Ceruloplasmin
CRABP1	Cellular retinoic acid-binding protein 1
DBI	Diazepam binding inhibitor
FABP5	Fatty acid-binding protein 5
FHL1	Four and a half LIM domains 1
FLNA	Filamin A
FTH1	Ferritin heavy chain 1
GLUT1	Glucose transporter 1
HBB	Haemoglobin subunit beta
HSPA5	Heat shock protein family A member 5
IL6	Interleukin 6
ITGAV	Integrin subunit alpha V
ITGB1	Integrin subunit beta 1
MIF	Macrophage migration inhibitory factor
MYH9	Myosin heavy chain 9
NAALAD2	N-acetylated alpha-linked acidic dipeptidase 2
PARK7	Parkinsonism-associated deglycase
PRDX2	Peroxiredoxin 2
PYGL	Glycogen phosphorylase L
RHO	Rhodopsin
SLC2A1	Solute carrier family 2 member 1
SLC12A2	Solute carrier family 12 member 2
SLC25A5	Solute carrier family 25 member 5
SPP1	Secreted phosphoprotein 1
STOM	Stomatin
STRA6	Signalling receptor and transporter of retinol STRA6
TAGLN	Transgelin
TMSB10	Thymosin beta 10
TSPAN14	Tetraspanin 14
HREC	Human Research Ethics Committee
NES	Normalised enrichment score
LDH	Lactate dehydrogenase
