# Supplementary material for: Region-Specific Proteomic Profiles of Extracellular Vesicles (EVs) Derived from Human Macular and Peripheral RPE-Choroid Explants
Source: Biomedicines. 2026 Jul 30;14(8):1715. doi: 10.3390/biomedicines14081715 (PMC13509229; doi:10.3390/biomedicines14081715)
Supplement: Supplementary file 1 [file biomedicines-14-01715-s001.zip › Figure S1 - PLS-DA models _ KEGG-GSEA pathway _ LDH assay.pdf]

biomedicines

MDPI

Supplementary Figure 1 (Figure S1)

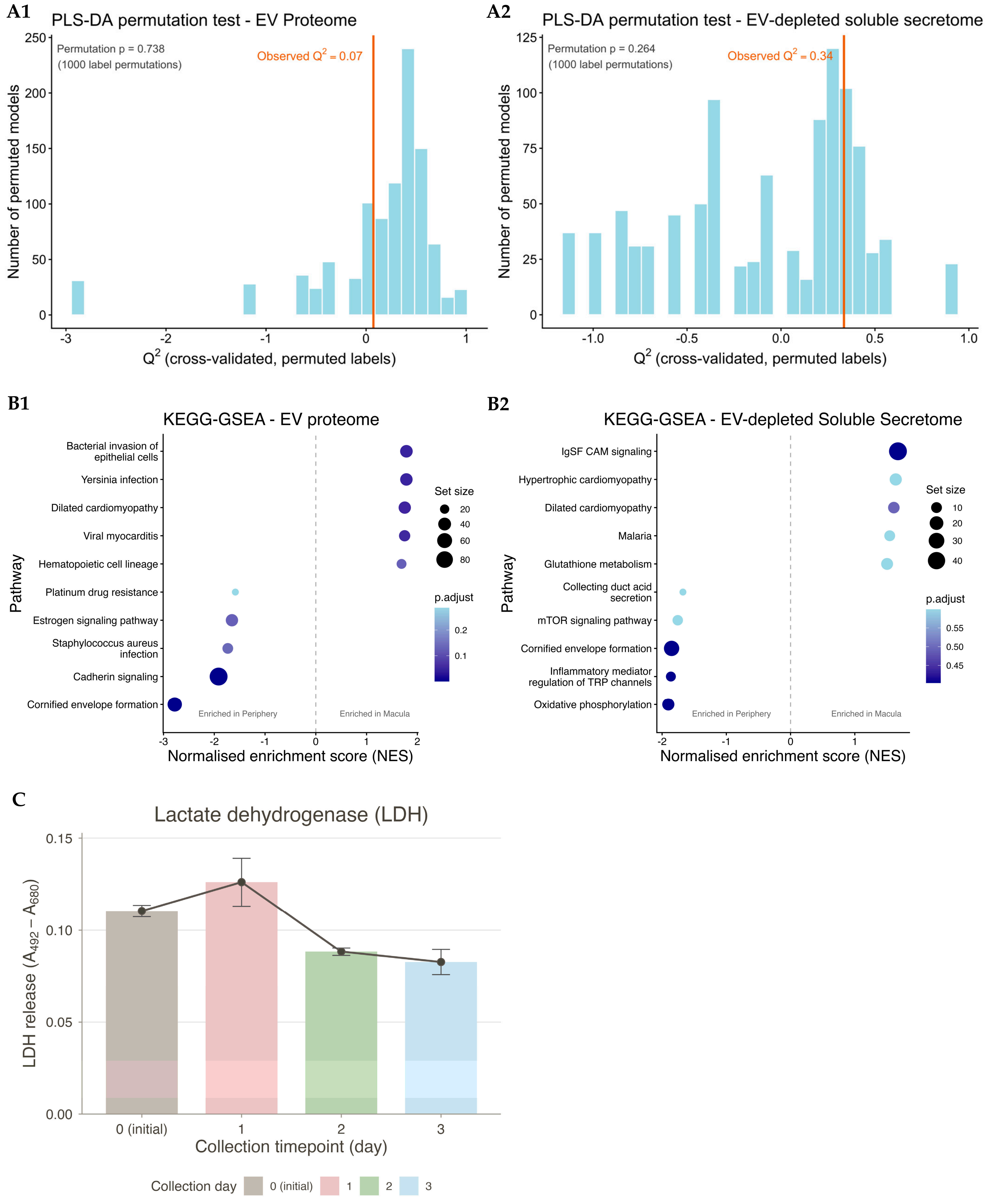

Figure S1. Permutation testing of PLS-DA models, KEGG-GSEA pathway of the EV proteome and EV-depleted soluble secretome, and LDH release assay of RPE-choroid explant viability. (A1, A2) Permutation testing of the PLS-DA models for the EV proteome (A1) and EV-depleted soluble secretome (A2). Histograms show the distribution of cross-validated Q^2 values obtained from 1000 random permutations of the group labels (light blue bars), and the orange vertical line indicates the observed Q^2 of the model trained on the true group labels. The observed Q^2 and permutation-derived p-value were 0.07 and 0.738 for the EV proteome (A1) and 0.34 and 0.264 for the EV-depleted soluble secretome (A2), respectively. **(B1, B2)** KEGG-GSEA of the EV proteome (B1) and EV-depleted soluble secretome (B2). GSEA was performed on the full ranked list of all quantified proteins, ranked by $\log_2$ fold-change between macular and peripheral samples, without applying a differential-abundance cut-off. For each dataset, the pathways with the five most positive and five most negative normalised enrichment scores (NES on x-axis) are shown. A positive NES indicates enrichment in the macular samples and a negative NES enrichment in the peripheral samples (dashed line, NES = 0). Dot size represents the gene-set size, and dot colour represents the adjusted p-value (FDR). **(C)** Lactate dehydrogenase (LDH) release assay assessing RPE-choroid explant viability. LDH release, expressed as reference-corrected absorbance ($A_{492} - A_{680}$), was measured in one hour conditioned medium at baseline time point (day 0) and on successive culture days (1–3). Bars show the mean of technical triplicates, the overlaid line traces the release trajectory, and error bars represent ± SD.
